# Identification of a SARS-CoV-2 Lineage B1.1.7 Virus in New York following Return Travel from the United Kingdom

**DOI:** 10.1128/MRA.00097-21

**Published:** 2021-03-04

**Authors:** Leonardo C. Caserta, Patrick K. Mitchell, Elizabeth Plocharczyk, Diego G. Diel

**Affiliations:** a Department of Population Medicine and Diagnostic Sciences, Animal Health Diagnostic Center, College of Veterinary Medicine, Cornell University, Ithaca, New York, USA; b Cayuga Medical Center at Ithaca, Ithaca, New York, USA; Queens College

## Abstract

Here, we report the identification and coding-complete genome sequence of a severe acute respiratory syndrome coronavirus 2 (SARS-CoV-2) strain (NYI.B1-7.01-21) obtained from a patient with symptoms of COVID-19 who had a recent travel history to the United Kingdom. The sample was tested by the Cayuga Health Systems laboratory as part of New York State’s travel testing guidance and was sequenced at Cornell University after testing positive.

## ANNOUNCEMENT

Severe acute respiratory syndrome coronavirus 2 (SARS-CoV-2), a member of the genus *Betacoronavirus*, family *Coronaviridae* (1), was first identified in Wuhan, China (2). Since its emergence in December 2019, several mutations have been detected, leading to the emergence of multiple genetic lineages. The lineage B.1.1.7 was first detected in September 2020. It is characterized by a large number of mutations and has since been detected in numerous countries around the world (3). As of 25 January 2021, 22 cases of this strain have been identified in the state of New York (4).

The sample characterized here was obtained for routine diagnostics for SARS-CoV-2 on 7 January 2021 from a person returning from a trip to England and presenting symptoms of shortness of breath, fatigue, and fever (institutional review board [IRB] approval number CMC0420EP). Viral RNA was extracted from a saliva sample treated with inactivating medium (containing guanidine hydrochloride) using the QIAamp viral RNA minikit (Qiagen). The sequencing library was prepared for sequencing on an Oxford Nanopore Technologies (ONT) MinION instrument using a modification of the approach developed by the ARTIC Network for sequencing SARS-CoV-2 (https://www.protocols.io/view/ncov-2019-sequencing-protocol-v2-bdp7i5rn). A total of 58 primers were designed using Primer3 (5) in the Geneious Prime 2019 software targeting approximately 1,500-bp and 500-bp products with 100 bp of overlap between different amplicons. Primer sequences are available at dx.doi.org/10.17504/protocols.io.brkxm4xn, and the first-strand synthesis and PCR conditions are available at dx.doi.org/10.17504/protocols.io.br54m88w. Libraries were multiplexed and sequenced on an R9.4 flow cell for 6 h.

Raw reads were base called and demultiplexed with the MinIT device (ONT) and then processed through the artic-ncov2019-medaka conda environment (https://github.com/artic-network/artic-ncov2019). We obtained a 29,713-bp genome with a mean depth of coverage of 2,236.3× and a GC content of 38%. The sequence does not cover 51 bp from the 5′ end and 121 bp from the 3′ end of the reference genome (GenBank accession number NC_045512.2). Mutations were also verified in Geneious, and a table of variations in relation to the reference genome was generated (Table 1).

**TABLE 1:** Mutations of SARS-CoV-2 strain NYI.B1-7.01-21 in comparison to the reference strain (GenBank accession number NC_045512.2)

Gene or region	Nucleotide position	Amino acid change	CDS codon position^*a*^	Nucleotide change
5′ untranslated region	241			C → T
ORF1ab	913		216	C → T
	2110		615	C → T
	2485		740	C → T
	3037		924	C → T
	3267	T → I	1001	C → T
	5388	A → D	1708	C → A
	5986		1907	C → T
	6954	I → T	2230	T → C
	7984		2573	T → C
	11288–11296	SGF deletion	3675–3677	
	14120	P → L	4619	C → T
	14408	P → L	4715	C → T
	14676		4804	C → T
	15279		5005	C → T
	16176		5304	T → C
	19390	P → S	6376	C → T
S	21765–21770	HV deletion	69−70	
	21991–21993	Y deletion	144	
	23063	N → Y	501	A → T
	23271	A → D	570	C → A
	23403	D → G	614	A → G
	23604	P → H	681	C → A
	23709	T → I	716	C → T
	24506	S → A	982	T → G
	24914	D → H	1118	G → C
ORF3a	25638		82	C → T
ORF8	27972	Q → stop	27	C → T
	28048	R → I	52	G → T
	28095	K → stop	68	A → T
	28111	Y → C	73	A → G
N	28280–28282	D → L	3	GAT → CTA
	28881–28883	RG → KR	203–204	GGG → AAC
	28977	S → F	235	C → T

a CDS, coding DNA sequence.

**FIG 1:**
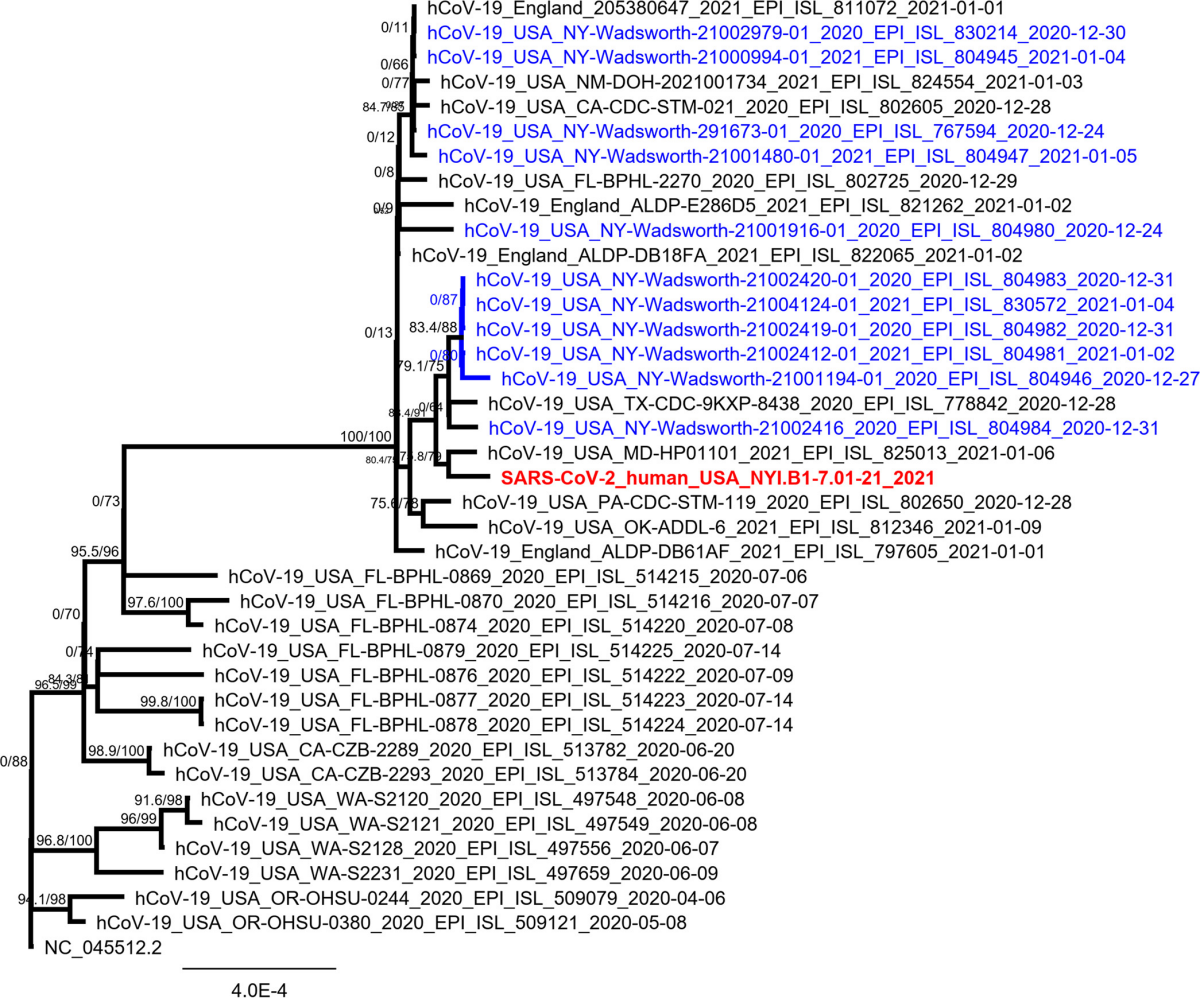
Phylogenetic tree of strain NYI.B1-7.01-21, highlighted in red in a clade with other B.1.1.7 sequences. Other samples from New York belonging to this lineage are shown in blue. A whole-genome nucleotide sequence alignment was performed with sequences retrieved from GISAID (https://www.gisaid.org) using MAFFT v7.450 (8) with the parameters Auto for algorithm, scoring matrix of 200PAM/k=2, gap open penalty of 1.53, and offset value of 0.123. The phylogenetic tree was constructed using 1,000 bootstrap replicates and the model TIM2+F, selected as the best-fit model by the IQ-TREE Web server (9).

Phylogenetic analysis classified strain NYI.B1-7.01-21 as part of the B.1.1.7 lineage (Fig. 1). Compared to the reference sequence (GenBank accession number NC_045512.2), a total of 34 mutations and deletions were detected, including all the nonsynonymous mutations inferred to occur on the branch leading to the B.1.1.7 lineage. An additional mutation at position 28881 to 28883, GGG > AAC (R203K and G204R), in the N gene may have impacts on the structure and function of the N protein (6, 7). Open reading frame 8 (ORF8) has an additional stop codon, downstream of the Q27 stop. Our analyses show that SARS-CoV-2 NYI.B1-7.01-21 does not cluster with the other B.1.1.7 viruses detected in the state of New York to date, indicating an independent introduction. These observations are consistent with the patient’s travel history to England and highlight the importance of arrival testing and genetic characterization of SARS-CoV-2-positive samples, especially following travel to locations where newly emerging SARS-CoV-2 variants are known to be circulating.

### Data availability.

This sequence has been deposited in GenBank under the accession number MW487270. The accession numbers for the raw sequencing reads in the NCBI Sequence Read Archive (SRA) are PRJNA692972 (BioProject), SAMN17373206 (BioSample), and SRR13453793 (SRA).
